# Multiple Complicated Concurrent Hernias in a Single Patient: A Case Report

**DOI:** 10.7759/cureus.56582

**Published:** 2024-03-20

**Authors:** Huy Q Nguyen, Toan K Dang, Hien T Tran, Huy L Phan, Dang Khoa D Ho

**Affiliations:** 1 Department of General Surgery, People's Hospital 115, Ho Chi Minh, VNM

**Keywords:** abdominal hernias, laparoscopic hernia repair, paraesophageal hernias, obturator hernias, multiple complicated concurrent hernias

## Abstract

Multiple complicated concurrent hernias with obturator hernia and paraesophageal hernia unusually occur in clinical settings. The obturator hernias belong to a rare pelvic hernia that accounts for a minority of all abdominal hernias. Besides, paraesophageal hernias occur commonly in elderly female patients. Clinical manifestations of these hernias are usually unspecific and the diagnosis is based on computed tomography (CT). In this paper, we presented a case of multiple complicated hernias in an 81-year-old woman. She was admitted to our hospital due to intestinal obstruction that was caused by a simultaneous obturator and paraesophageal hernia. She was successfully treated by laparoscopic hernia repair. Postoperative progression was favorable. She was then discharged from the hospital after four hospital days.

## Introduction

Hernia is defined as the condition of organs herniated to protrude outside the normal limits of the abdominal cavity, under the skin, or through an acquired or congenital weakness of the abdominal wall [1]. Hernias are classified according to causes, localization, anatomy, or clinical manifestation [1]. The worldwide incidence of abdominal wall hernias was roughly 4%-5% [2].

An obturator hernia is an uncommon external abdominal wall that accounts for <1% of all abdominal wall hernias and mainly occurs in thin female patients between the ages of 70 and 90, having given birth many times [3]. This commonly occurs on the right side because the sigmoid colon might cover the left obturator foramen [4]. Preoperative diagnosis of obturator hernia is a challenge due to non-specific symptoms, usually only discovered during surgery due to bowel obstruction or peritonitis [4,5]. That leads to an increase in mortality and postoperative complications [4]. Several signs might indicate this disease such as Howship-Romberg (pain along the inner thigh and extends to the knee on internal rotation of the hip) and Hannington-Kiff sign (the absence of adductor reflex in the thigh while patellar reflex is intact) [5].

A hiatal hernia is a protrusion of abdominal anatomy into the thoracic cavity via the hiatus opening [6]. Paraesophageal hernia accounted for 5% to 15% of all hiatal hernias, which manifest gastric volvulus due to the loosening of the peritoneal attachments of the stomach and subsequent rotation of the gastric fundus [6]. Hiatal hernias were classified into four types: type I sliding hernia is a sliding hernia (95% of all hiatal hernias) in which the gastroesophageal (GE) junction: the junction of the distal esophagus and stomach cardia) herniates above the diaphragm, type II pure paraesophageal is a paraesophageal hiatal hernia, which occurs when part of the stomach rolls up to the diaphragm through the esophagus, type III is a mixture of type I and II with the herniation of both the fundus and gastroesophageal, type IV is any structure other than the stomach herniating through the hiatus [7]. The risk factors are age, trauma, surgical history, and heredity contributing to the increase in the prevalence of paraesophageal hernia [8]. The surgical approach is recommended to treat all symptomatic paraesophageal hernias and asymptomatic larger hernias in a healthy patient under 60 years old [9].

In this paper, we presented a rare case of an 81-year-old female patient suffering from small intestine obstruction due to concurrent hernias of the obturator and paraesophageal hernia. The patient was treated successfully by laparoscopic hernioplasty at People’s Hospital 115.

## Case presentation

The 81-year-old female patient presented to our hospital with the chief complaint of abdominal pain. Her medical history recorded open cholecystectomy 10 years ago, senility, and gastroesophageal reflux disease (GERD). The disease’s onset began three days before the hospital admission. The patient reported dull pain in the umbilical region and two sides of the lumbar region. The pain gradually increased and was accompanied by nausea. One day before admission, the pain was intense and she vomited after eating.

**Figure 1:**
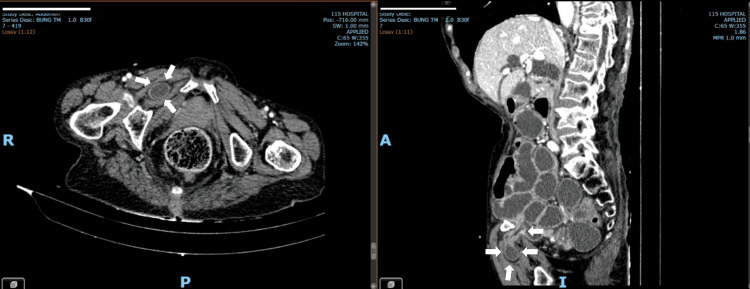
Images of the obturator hernia Axial and sagittal CT scans after contrast injection revealed intestinal obstruction at the transition point in the right iliac fossa due to the obturator hernia (the arrows).

**Figure 2:**
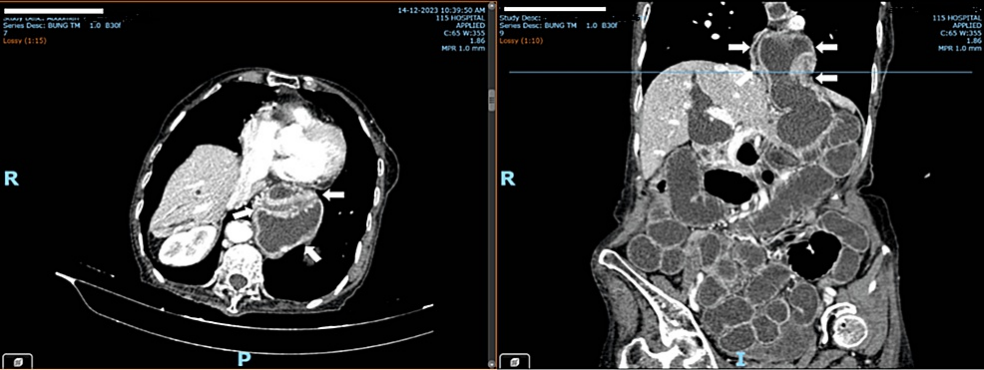
Images of the paraesophageal hernia CT scan of the axial and coronal planes after intravenous contrast injection detected gastric herniation through the esophageal opening (the arrows).

The patient was admitted to the Emergency Department in a state of alertness with stable vital signs and a thin and malnourished condition. Physical examination revealed a mild abdominal distention with periumbilical tenderness. The blood tests, including complete blood count and routine blood biochemistry, were normal. The abdominal ultrasound suggested the state of intestinal obstruction and intrahepatic as well as extrahepatic bile duct dilatation. The patient was under monitoring in the Department of General Surgery. Here, she received complete parenteral nutrition. However, during follow-up, the patient’s condition did not improve. One day later, a contrast abdominal computed tomography (CT) scan detected that intestinal obstruction at the transition point in the right iliac fossa showed due to an obturator hernia, the intestinal loops were still enhancing, and no thrombosis of the superior mesenteric vein was seen. At the same time, gastric herniation through the gastroesophageal opening, large dilation of the stomach in this position (Figures 1, 2). Clinical symptoms at this time were little changed compared to when admitted to the hospital, vomiting decreased, abdominal periumbilical pain, inguinal and anterior thigh hernias were not palpable after exercise testing, Howship-Romberg sign (±), Hannington-Kiff (-).

Finally, the diagnosis was intestinal obstruction due to an obturator hernia and a hiatus hernia of the esophagus with dilatation of the bile duct of unknown cause. We applied a laparoscopic approach, though it was difficult to enter the abdomen due to an old incision, and noted the greater omentum and intestinal loops attached to the old incision. After removing all adhesions, we explored the right iliac fossa, one meter from an ileocecal angle, there was a segment of the intestine entering the obturator foramen of the lateral wall. The segment above the transitional point was completely dilated and had fluid retention. We released this segment from the obturator canal, and it returned better blood perfusion. After that, we restored the obturator orifice with Silk 2/0. An examination of the upper abdominal part noted the cardia and gastric fundus protruding into the mediastinum through the esophageal opening along with the hernia sac (type III hiatal hernia). We conducted the dissection of the hernia sac and pulled all the viscera into the abdomen. Then, we continued to perform laparoscopic hernioplasty: simple cruroplasty without mesh reinforcement and rolling the fundus in Nissen’s fundoplication to prevent recurrence and gastroesophageal reflux.

Postoperatively, the progress of the patient was favorable; she had flatus and could eat 24 hours later. After that, she was discharged from the hospital on the fourth day after surgery in stable condition.

## Discussion

In this paper, we reported a very rare case of multiple hernias, including an obturator hernia and a hiatal hernia concurrent in a single patient. Multiple concomitant abdominal hernias are very unusual [10]. There were only several documents of individual cases with simultaneous hernias such as bilateral inguinal and femoral hernias, occult and groin hernia, concomitant bilateral inguinal, femoral, and obturator hernias, ... [10-13]. These cases usually occur in geriatric patients and involve multiple abdominal wall defects. An obturator hernia is an unusual abdominal wall hernia with an incidence of 0.07-1%, occurring in the obturator canal due to the weakening of the pubic fascia of the obturator membrane between the ischium and pubic bones [14]. An obturator hernia is often called a “petite older women's hernia” [14]. Our patient's characteristics are consistent with previous reports that obturator hernia is common in the elderly, chronically ill, multiparous, and malnourished women with multiple comorbidities. The most common signs and symptoms of obturator hernia include bowel obstruction, abdominal pain, groin pain, and a palpable groin mass [12]. Patients may also have the Howship-Romberg sign, associated with inner thigh pain when internally rotating the hip; and the Hannington-Kiff sign; characterized by loss of adductor reflexes [15]. However, clinical symptoms are rarely sufficient and specific to confirm the diagnosis. Recent reports emphasize the role of CT in diagnosing obturator hernia [16,17]. Ho-Hsing Lin et al. reported that the preoperative diagnosis rate improved from 43% to 90% because of CT [16]. In fact, with our patient, we also discovered an additional disease: paraesophageal hernia through an abdominal CT Scan. This hernia often has no typical symptoms and is often diagnosed when discovered incidentally on CT scans or emergency conditions such as intestinal obstruction [7]. Several common symptoms are heartburn, chest pain, difficulty swallowing, feeling of bloating after eating, shortness of breath, and cough [7]. In our patient, during laparoscopic surgery, revealing at the obturator hernia site, we found the wall of the ileum 1 meter from the ileocecal root entering the right lateral obturator canal that suggested a Richter's hernia. This is a type of hernia in which only a position of the intestinal lumen is herniated through fascial defects [17]. The most common hernia component is the terminal ileum. Several authors reported the obturator hernia was more dominant on the right side, which also corresponds to the location of the ileum [4].

With the development of laparoscopic equipment, the obturator hernia can be treated by a laparoscopic approach. Only in some conditions, such as extensive inflammation, adhesions, anatomical variations, diabetes mellitus, obesity, metabolic syndrome, etc., open approaches are more advantageous [18]. The prevalence of bowel resection in obturator hernias was 25-50% [15]. In our case, after releasing the intestinal segment from the obturator canal, we assessed good blood perfusion and decided to preserve it. Restoration of the abdominal wall can be performed with or without an artificial mesh. Most patients achieved good results after primary hernia repair without mesh placement, but the recurrence rate may increase [19]. If the obturator foramen was not restored, the reported recurrence rate would be approximately 10% [19]. In our report, the obturator hernia was released and the hernia was repaired by directly suturing the obturator foramen with a Silk 2/0 suture.

During the exploration, the gastric cardia and fundus were noted through the esophageal opening into the chest. Therefore, it was classified as a type III hiatal hernia. A hiatal hernia can also occur congenitally due to a defect in the esophageal hiatus in the diaphragm or due to congenital shortening of the esophagus that pulls the stomach upward [20]. These hernias are also uncommon in clinical practice; however, they occurred concomitantly in a single patient, which was extremely rare. Paraesophageal hernia repair can performed with mesh reinforcement or without [21]. Oppelt et al. suggested that the use of a mesh should be applied to cases of large diaphragmatic defects [22]. While mesh reinforcement in laparoscopic hiatal hernia repair demonstrated early success at reducing recurrence rates, several limitations emerged. In 2002, a randomized controlled trial compared mesh reinforcement to the suture cruroplasty with only cruroplasty. They reported that the simple cruroplasty group had a recurrence rate of 22% after one year, compared with 0% in the combination group [23]. In this case, the gastric fundus was released from the esophageal opening, and the entire part was pulled down to the abdomen. We just conducted a hernioplasty and rolled the fundus following Nissen’s fundoplication.

## Conclusions

In this case, we presented an extremely rare case of simultaneous double hernias, including obturator and paraesophageal hernias. Each of these hernias is also an unusual hernia in clinical settings that is only detected when there are complications such as intestinal obstruction. The laparoscopic approach is minimally invasive and could treat successfully these multiple complicated hernias.
